# Which nutritional prognosis is better? comparison of the three most commonly performed bariatric surgeries: A systematic review and network meta-analysis

**DOI:** 10.3389/fsurg.2022.1065715

**Published:** 2023-01-30

**Authors:** Yuanyao Cui, Di Zhang, Li Wang, Xuefei Liu, Chunyan Wang, Shuyun Tian, Meiqu Li

**Affiliations:** ^1^ Department of Nursing, School of Medicine and Nursing, Dezhou University, Dezhou, China; ^2^ Department of Oral Medicine, Binzhou Medical University Hospital, Binzhou, China; ^3^ Department of Stomatology, Binzhou Medical University, Binzhou, China

**Keywords:** bariatric surgeries, malnutrition, obesity, micronutrients, RYGB, LAGB, SG

## Abstract

**Background:**

Obesity is one of the most important public health conditions in the world, and surgical intervention is the only medical treatment recognized by the medical community as a complete and permanent cure for morbid obesity and its complications. The choice of surgical modality is also based more on the experience of the physician or the requirements of people with obesity, rather than on scientific data. In this issue, a thorough comparison of the nutritional deficiencies caused by the three most commonly used surgical modalities is needed.

**Objectives:**

We aimed to use the network meta-analysis to compare the nutritional deficiencies caused by the three most common BS procedures in many subjects who underwent BS to help physicians determine the best BS surgical approach to apply to their clinical people with obesity.

**Setting:**

A systematic review and network meta-analysis of world literature.

**Methods:**

We followed the Preferred Reporting Items for Systematic Reviews and Meta-Analyses, systematically reviewed the literature, and conducted a network meta-analysis using R Studio.

**Results:**

For the four vitamins calcium, vitamin B12, iron and vitamin D, the micronutrient deficiency caused by RYGB is the most serious.

**Conclusions:**

RYGB causes slightly higher nutritional deficiencies in Bariatric surgery, but RYGB remains the most commonly used modality for Bariatric surgery.

**Systematic Review Registration:**

https://www.crd.york.ac.uk/prospero/display_record.php?ID=CRD42022351956, identifier: CRD42022351956.

## Introduction

Obesity is one of the world's most important public health conditions (1). According to the World Health Organization, the global obesity rate has tripled since 1975 (2). Due to rising population numbers and an aging population, in 2019, obesity is responsible for approximately 5.02 million deaths from non-communicable diseases and 102 million disability life-adjusted years, equivalent to 12% of all non-communicable disease deaths (3). Surgical intervention is the only medical treatment recognized by the medical community to completely and permanently treat morbid obesity and its complications (4). Surgical interventions can reduce weight and improve obesity complications through gastrointestinal surgery, achieving a good prognosis (5).

Bariatric surgery (BS) can be divided into two general types: malabsorptive (bypassing certain parts of the gastrointestinal tract to reduce the area of absorption) and restrictive (reducing the gastric volume to allow people with obesity to achieve satiety quickly) (6). Malabsorptive types include Roux-en-Y gastric bypass (RYGB) and biliopancreatic duodenal transposition (BPD), and restrictive types include laparoscopic adjustable gastric banding (LAGB) and laparoscopic sleeve gastrectomy (SG). The three most widely used types in clinical practice are SG, RYGB, and LAGB (7).

However, BS also has its disadvantages. Because surgery alters parts of the gastrointestinal tract, it often results in micronutrient deficiencies (8). Some of these nutrient deficiencies can have severe clinical consequences, including various complications such as Wernicke's encephalopathy and pediculosis due to vitamin B1 deficiency, iron deficiency anemia due to iron deficiency, megaloblastic anemia due to vitamin B12 or folic acid deficiency, bone mineralization and fracture risk due to vitamin D or calcium deficiency, etc (9). In addition to these severe complications, many case reports have reported people with obesity with nutritional deficiencies after BS, but few studies have clarified their incidence after different BS surgical approaches (10). In the published studies, either only one or two BS procedures were evaluated for comparison, only a small number of subjects were included, or the nutritional assessment was not exhaustive (11). The choice of surgical modality was also based more on physician experience and people with obesity requirements than on scientific data. A comprehensive comparison of the nutritional deficiencies caused by the three most commonly used surgical approaches is needed on this topic.

Therefore, we conducted a network meta-analysis of the published literature on BS and nutritional deficiencies. We aimed to use the network meta-analysis to compare the nutritional deficiencies caused by the three most common BS procedures in many subjects who underwent BS to help physicians determine the best BS surgical approach to apply to their clinical people with obesity.

## Method

### Criteria for consideration of study inclusion

We included studies that should have compared the deficiency rates and complication rates of 4 nutrients (vitamin B12, iron, vitamin D, and calcium) in people with obesity after different BS surgical approach manuscript Formatting. Prior to the start of the study, it was registered in the International Prospective Register of Systematic Reviews (PROSPERO) (CRD42022351956).

### Search methods

We followed the Preferred Reporting Items for Systematic Reviews and Meta-Analyses (PRISMA) (12). Our search strategy aimed to identify all studies examining the impact of different BS surgical approaches on people with obesity nutrient deficiencies. We used Mesh and did our best to include all synonymous medical terms in the search to avoid omissions. Two co-first authors collaborated with a Ph.D. (the second author of this paper) to identify search terms, as well as relevant search terms and keywords for our primary outcome, nutrient deficiency. We also identified search criteria and strategies and searched PubMed, Embase, Scopus, Google Scholar, and the Cochrane Clinical Trials Register for eligible studies. To ensure comprehensiveness, we also searched the cited literature of relevant reviews on an article-by-article basis.

### Data selection

Several inclusion criteria were used in determining study eligibility.

a. Study design: non-review, non-letters, and other non-research articles.
b. Participants: people with obesity who underwent BS surgery.
c. Interventions and comparisons: studies comparing nutritional deficiencies after two or more BS surgical approaches.
d. Results: these studies reported nutrient deficiency rates as well as complication rates.
e. Data: the data included in the study were not duplicated.
f. Language: the language used in the studies was English.

The two co-first authors performed data searches in various databases and performed initial screening based on the review of titles and abstracts to determine study eligibility. All references were imported into Zotero and automatically screened for duplicates by this software. Once we identified studies for potential inclusion, we screened the full text of the articles. Disagreements were resolved through discussion; if consensus could not be reached, the second author served as the arbiter. We also searched for disease guidelines, systematic reviews, and survey articles involving nutritional deficiencies after BS or BS and reviewed their reference sections to ensure that no studies were missed. When necessary, the three reviewers discussed any issues related to study inclusion or exclusion with each other and with the senior author. We used Zotero's notes feature to mark the general content of studies or reasons for exclusion to facilitate our full-text review. The manuscript did not involve the use of animal or human subjects.

### Data extraction

All three reviewers were involved, and many abstracts were identified for full-text review. All reviewers received training on study eligibility criteria and data extraction. A panel of three reviewers reads and assessed all included full-text articles to determine whether they met the eligibility criteria and, if not, to explain the reasons for exclusion. One reviewer independently extracted the following data elements from each article that met the eligibility criteria table developed by the study team: first author's name, year of publication, journal name, location, BS program model of the study, nutrient deficiency status, study population (including disease), selection of study cohort, sample size, study purpose, primary outcome, and statistical significance (if applicable) (13).

### Data synthesis and analysis

The study used the gemtc package in R (R Studio 2022.02.1 Build 461-Inc version) (https://posit.co) to analyze the data (14, 15). We performed a network meta-analysis (Markov chain Monte Carlo approach in a Bayesian framework) in order to study nutrient deficiencies due to different surgical modalities, pooling the different BS surgical modalities and nutrient deficiencies included in the studies (16). Not all studies could be included in the network meta-analysis, as some of the included studies did not include nutrient deficiency as a dichotomous variable and they only provided data for calculating odds ratios (ORs). We first developed a consistency model using forest plots to describe the comparison of ORs and 95% confidence intervals (CrI) between different BS surgical modalities. The difference in effect between interventions was judged to be statistically significant by whether the Crl of the interventions compared with each other crossed 1 or not. We also performed nodal analysis methods and consistency tests for all included studies to look for sources of heterogeneity and to ensure the assumption of homogeneity in the model.

In the study, we plotted trajectory and density plots to assess the volatility and overlap of MCMC chains and the convergence of the model (17). From the trajectory plots, we know whether the MCMC chain fluctuations are stable and have good overlap when the number of iterations reaches 5,000 or more; from the density plots, we know whether Bandwidth tends to 0 and reaches stability when the number of iterations reaches 20,000. When the requirements are satisfied, the synthesis indicates that the model converges well. We also plotted the Brooks-Gelman-Rubin diagnostic and calculated the potential size reduction factor (PSRF).

A satisfactory convergence model needs to satisfy two conditions at the same time:

a. 97.5% of the scaling factor and the median value of the scaling factor converge to 1 and reach stability after n iterations of calculation.
b. The PSRF value tends to be 1.

When the model satisfies both of the above conditions for convergence, we consider it a satisfactorily convergent model. The results of different BS surgical approaches were then taken and the probability of occurrence of a particular nutrient deficiency after different BS surgical approaches was deduced from the individual ranking results. The logarithms of the indices were then taken and compared with each other, and the results were plotted as bar charts. Although the ranking gives the ranking results for each BS surgical modality, it is not possible to simply conclude that the intervention is superior or inferior, but must be viewed in conjunction with other outcomes. We used the *I*^2^ test to assess heterogeneity. Because of the observed heterogeneity across studies, we used random effects models to estimate unadjusted and adjusted pooled rates and to identify sources of heterogeneity.

Final conclusions will be drawn from the following three sources:

a. from the forest plot as to which surgical modality was used with the highest risk of outcome events.
b. From the rank-ordering results, which intervention is ranked first in terms of probability of occurrence.

## Result

### Characteristics of included studies

**Figure 1:**
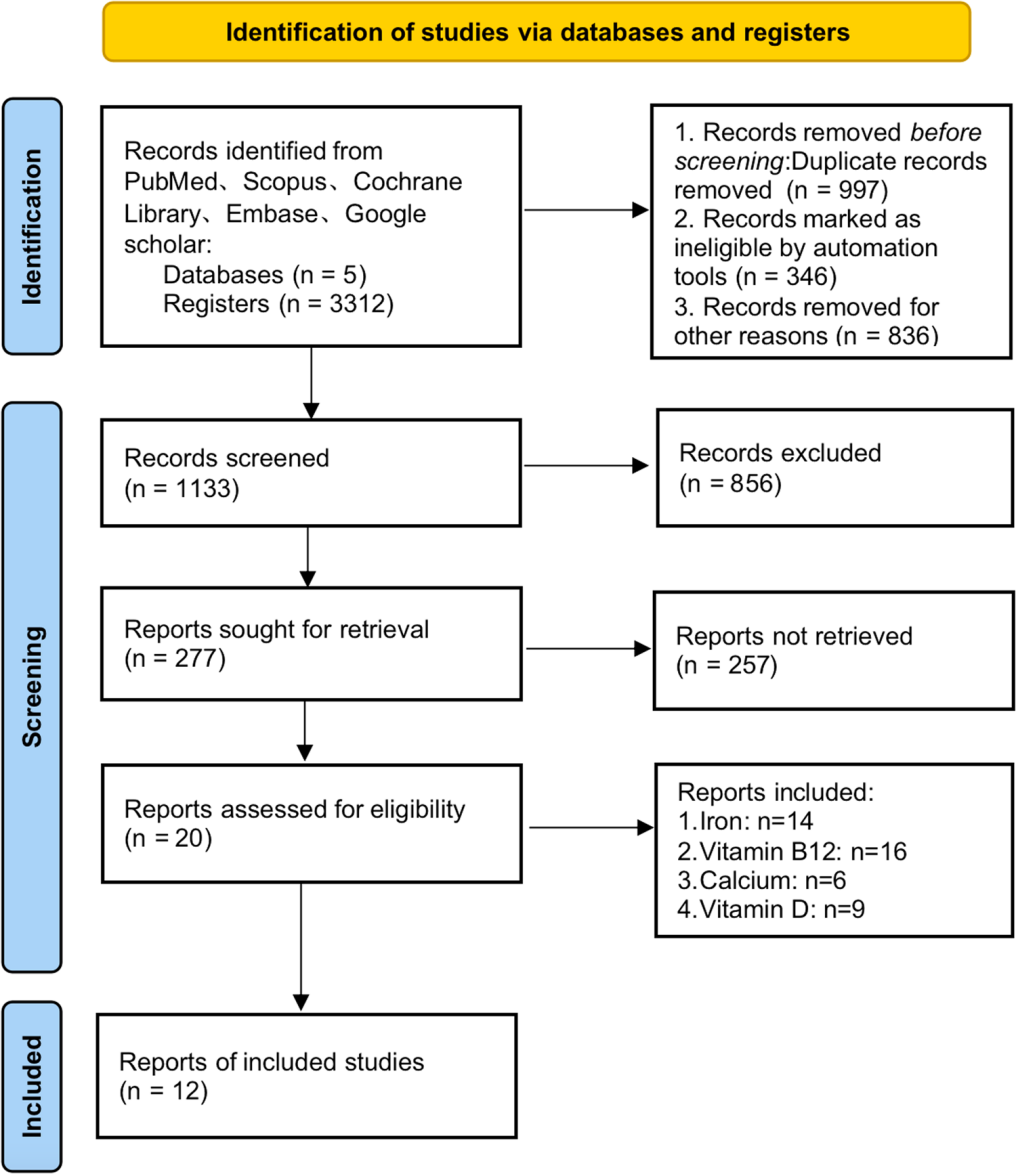
PRISMA flowchart.

3,312 potential studies were identified in our literature review (Figure 1).

**Figure 2:**
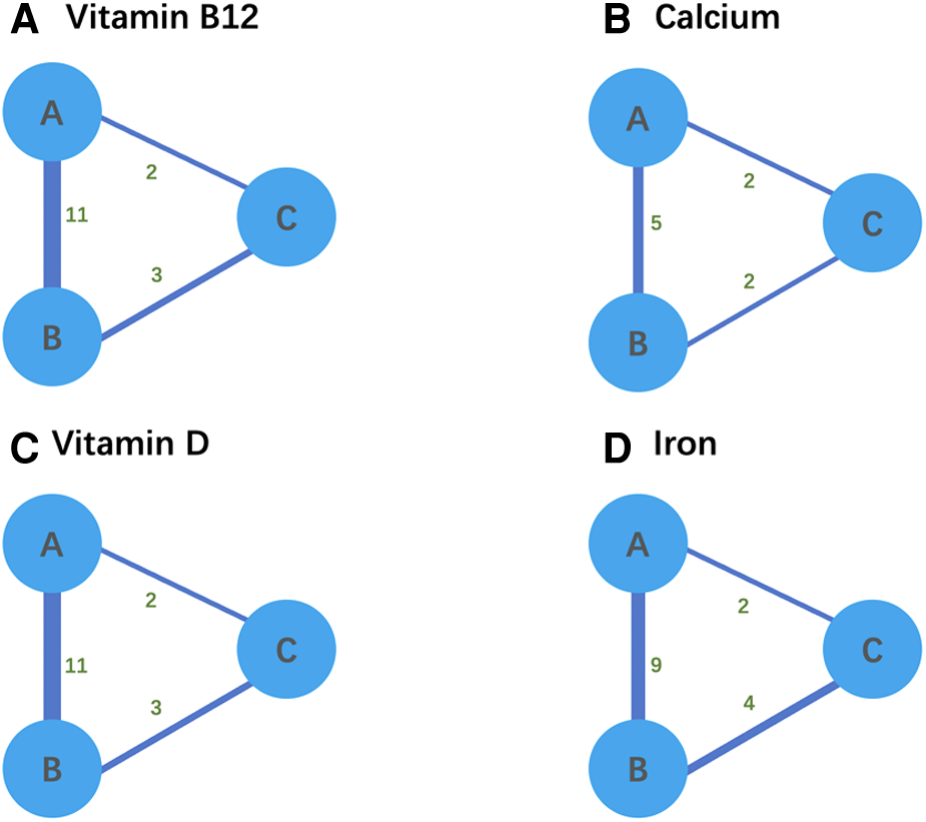
(**A–D**) Network relationship diagram for network meta-analysis. Thicker lines ndicate a greater cumulative number of enrolled studies per direct comparison. A refers to LSG, B refers to RYGB, and C refers to AGB.

We reviewed the citations, titles, and abstracts of 3,312 studies and identified 277 articles for inclusion. After a full-text review, 20 of these articles were included (Table 1). Studies were typically excluded because there was no comparison of surgical modalities or nutritional deficiencies were not the primary outcome. We began by mapping the network relationships in preparation for the network meta-analysis (Figure 2). While SG was directly compared with RYGB and LAGB due to the network linkages between trials in Figure 2, comparisons between the remaining two surgical modalities (i.e., RYGB and LAGB) were directly linked through at least two trials.

**Table 1:** Main characteristics of included studies.

Study	Size	Type of surgery	Nutrients
(18)	95	SG/RYGB	Iron/Vitamin B12/Vitamin D
(19)	98	SG/RYGB	Iron/Vitamin B12/Calcium
(20)	26	RYGB/LAGB	Iron/Vitamin B12
(21)	259,334	SG/RYGB/LAGB	Vitamin D
(22)	2,618	SG/LAGB	Iron/Vitamin B12
(23)	70	RYGB/LAGB	Iron/Calcium
(24)	576	SG/RYGB	Iron/Vitamin B12
(25)	89	SG/RYGB/LAGB	Vitamin D
(26)	136	SG/RYGB	Iron/Vitamin B12/Vitamin D
(27)	468	SG/RYGB	Vitamin B12/Calcium/Vitamin D
(28)	87	SG/RYGB	Iron/Vitamin B12
(29)	57	SG/RYGB	Iron/Vitamin B12/Vitamin D
(30)	494	SG/RYGB	Vitamin B12/Calcium
(31)	161	SG/RYGB	Vitamin B12/Vitamin D
(32)	52	SG/RYGB/LAGB	Iron/Vitamin B12/Calcium/Vitamin D
(33)	286	RYGB/LAGB	Iron/Vitamin B12
(34)	234	SG/RYGB/LAGB	Iron/Vitamin B12
(35)	52	SG/RYGB	Iron/Vitamin B12/Calcium
(36)	353	SG/RYGB	Iron/Vitamin B12
(37)	60	SG/RYGB/LAGB	Vitamin D

### Vitamin B12

**Figure 3:**
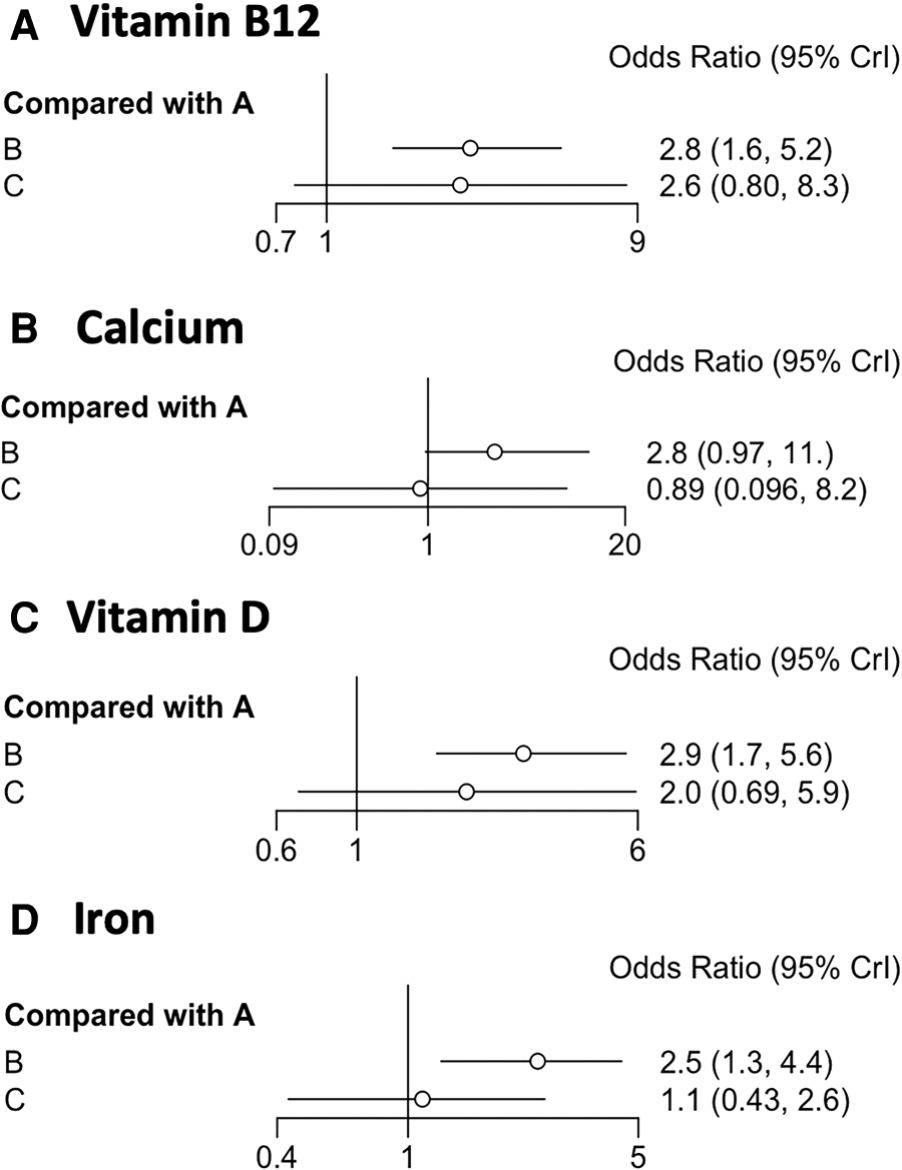
(**A–D**) Forest plots depicting OR values and 95% confidence intervals (CrI) for comparisons between operative BC and A. A refers to LSG, B refers to RYGB, and C refers to AGB.

We included 13 trials (*n* = 2408) that reported vitamin B12 deficiency after BS (Table 1). A network meta-analysis was used to rank the BS surgical modalities in order of frequency of postoperative vitamin B12 deficiency (from A–C). The forest plot in Figure 3 depicts A for SG, B for RYGB, and C for LAGB. We show the ORs and 95% CrI for the comparison by forest plot, where the OR for the outcome of intervention B compared with A was 2.90 and the OR for the outcome of C compared with A was 2.00, indicating that the risk of outcome events was 2.9 times higher for surgical modality B than for intervention modality A and 2 times higher for C. The 95% Crl for ORs were (1.70–5.60) and (0.68–5.80), respectively. Since the Crl of the intervention crossed 1 for C compared to A, the difference in effect between them was not statistically significant. The trajectory and density plots show that the model converged well and satisfied the required conditions.

**Figure 4:**
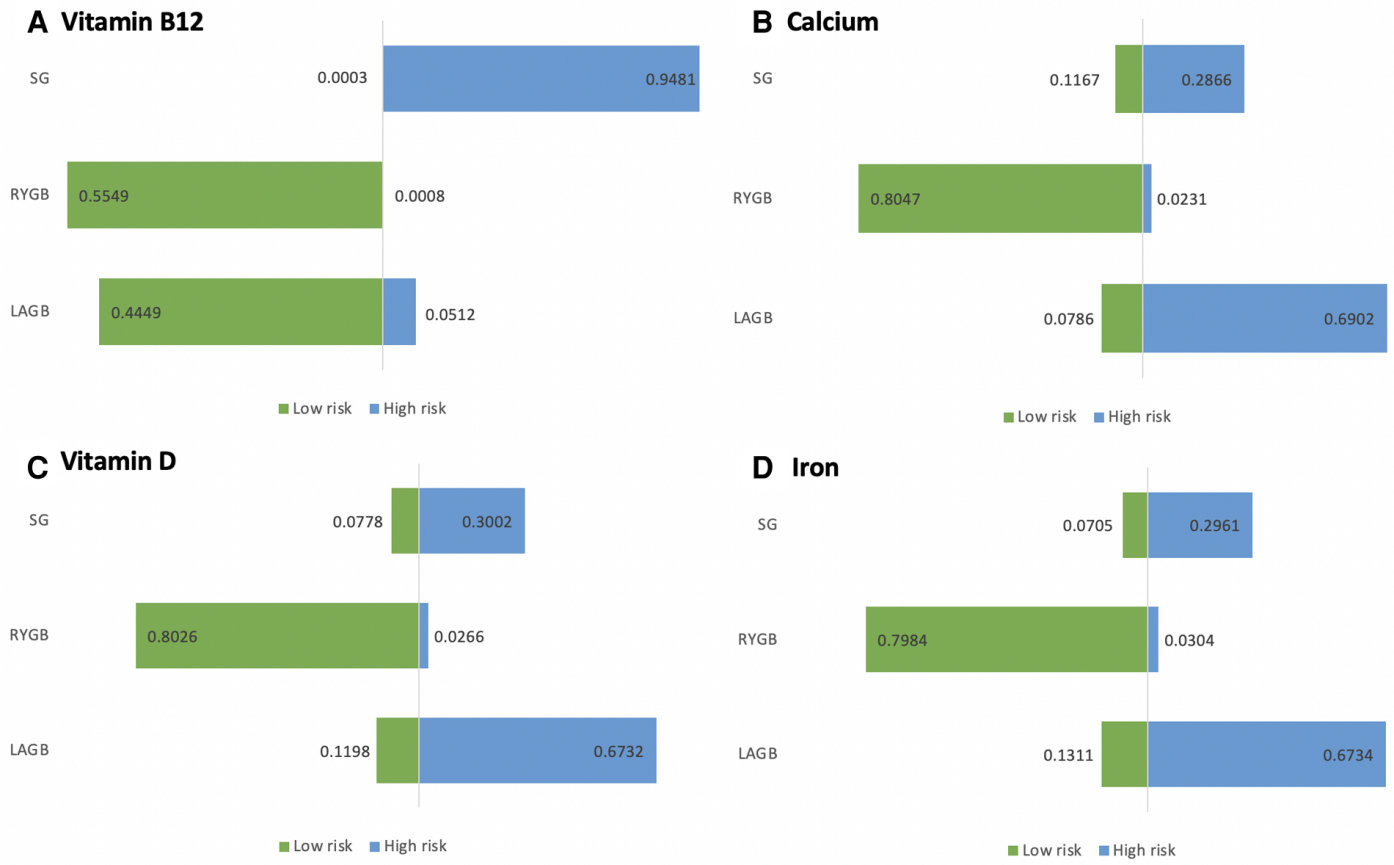
(**A–D**) Risk ranking chart risk probability ranking of nutrient deficiencies due to bariatric surgery.

We ranked the probability of vitamin B12 deficiency following BS for each of the three surgical procedures (see Figure 4A). Therefore, postoperative SG is better in terms of vitamin B12 absorption than the other two surgical approaches (i.e., RYGB and LAGB). Comparisons of RYGB with LAGB are not statistically significant.

### Calcium

Six of our included trials (*n* = 987) reported calcium deficiency after BS (Table 1). We ranked the frequency of postoperative calcium deficiency (from A–C). We show the ORs and 95% CrI for comparison by forest plot, with an outcome OR of 2.80 for intervention B compared to A and 0.89 for C compared to A. This indicates that the risk of an outcome event using surgical modality B is 2.8 times greater than the risk of an outcome event using intervention A and 0.89 times greater than the risk of an outcome event using intervention C. The 95% Crl for ORs was (0.97–11.00), (0.096–8.20). Since the Crl comparisons for the interventions all crossed 1, the difference in effect between them was not statistically significant. It can be seen from the trajectory and density plots that the model converged well and met the required conditions.

We compared each intervention A–C to each other and computed the logarithm of the index outcomes. For each of the three surgical procedures, the probability of postoperative calcium deficiency was ranked (see Figure 4B). As a result, we could only conclude from the ranked results that RYGB had a higher risk of postoperative calcium deficiency symptoms than SG and LAGB.

### Iron

We ranked the frequency of postoperative iron deficiency (from A–C). We show the ORs and 95% CrI of the comparisons by forest plots, with an OR of 2.50 for the outcome of comparison of intervention B with A and an OR of 1.10 for the outcome of comparison of C with A. This indicates that the risk of occurrence of an outcome event with surgical modality B is 2.5 times greater than the risk of occurrence of an outcome event with intervention A and 1.1 times greater with C. The 95% Crl of the ORs are (1.30–4.40), (0.43–2.60). Since the Crl for the comparison of A and C crossed 1, the difference in effect between them was not statistically significant. The incidence of RYGB is significantly higher than that of SG and LAGB as can be observed by the forest plot. it is clear from the trajectory and density plots that the model converges well and satisfies the required conditions.

The probability of postoperative calcium deficiency was ranked for each of the three surgical procedures (see Figure 4C). Direct comparison between interventions The *I*^2^ between pair-wise comparisons (pair-wise) was 0%, and the *I*^2^ between the results of network comparisons (network) was also 0%, then there was no heterogeneity between them and the homogeneity assumption was satisfied. Therefore, it can be judged that RYGB is more likely to cause iron deficiency symptoms in the postoperative period compared to SG and LAGB.

### Vitamin D

We ranked the frequency of postoperative vitamin D deficiency (from A–C). We showed the ORs and 95% CrI of the comparisons by forest plots, with an OR of 2.90 for the outcome of intervention B compared with A and 2.00 for the outcome of C compared with A. This indicates that the risk of an outcome event in surgical approach B is 2.9 times greater than the risk of an outcome event in intervention A and 2.0 times greater in C. The 95% Crl of the ORs was (1.7–5.60), (0.69–5.90). Since the Crl for the comparison of A and C crossed 1, the difference in effect between them was not statistically significant. From the forest plot, it can be seen that the incidence of RYGB is higher than SG and LAGB. from the trajectory and density plots, it can be seen that the model converges well and satisfies the required conditions.

The probability of postoperative calcium deficiency was ranked for each of the three surgical procedures (see Figure 4D). The direct comparison between interventions (pair-wise) is 0%, and the *I*^2^ between network comparison (network) results is also 0%, then there is no heterogeneity between them and the homogeneity assumption is satisfied. Therefore, it can be judged that RYGB is more likely to cause postoperative Vitamin D deficiency symptoms compared with SG and LAGB.

## Discussion

### The discussion

The goal of choosing the most appropriate surgical option is always to create the best balance between weight loss, complications, and micronutrient deficiencies. In clinical practice, BS is often performed only with SG, RYGB, and LAGB (38). Current data suggest that RYGB is more likely to cause micronutrient deficiencies than SG and LAGB. Although previous studies have compared the three most commonly used procedures, there is still a lack of more accurate evidence to choose the most scientific procedure.

Compared with RYGB, LAGB has a higher risk of slip/dilation and surgical reversal/conversion, but a lower risk of stricture, ulceration, and hernia compared with RYGB (39). LAGB has a shorter duration of stay compared to RYGB; SG has a higher risk of reoperation compared to RYGB (40). Compared with RYGB, SG and LAGB are associated with a higher failure rate or secondary surgery, which may be the main reason for the current high clinical use of RYGB.

**Figure 5:**
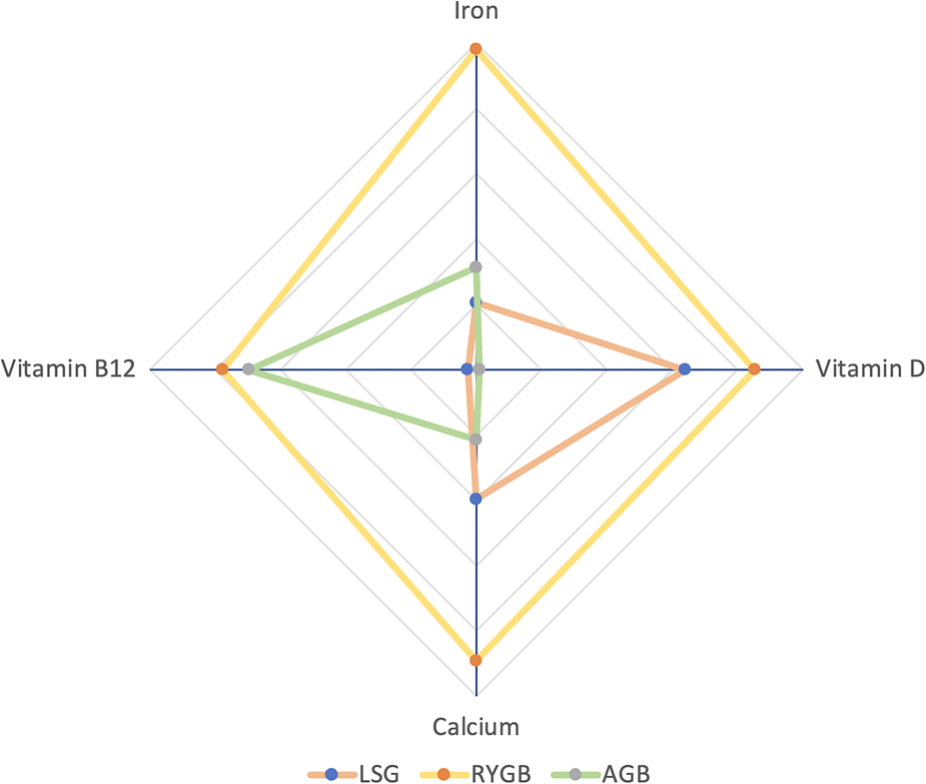
Rank-ordered radar chart: the farther from the center point is, the higher the probability of the corresponding event.

We used radar plots to show the combined ranking results of nutrient deficiencies (Figure 5). In Figure 5, there are three groups (i.e., SG, RYGB, and LAGB) with four dimensions (i.e., Vitamin B12, calcium, Vitamin D, and Iron). The farther from the center point means the higher risk of deficiency of that nutrient. The results showed that RYGB was more likely to cause vitamin B12 deficiency compared to SG, while LAGB was less pronounced, but LAGB was less deficient than RYGB (41). This may be because RYGB is a malabsorptive procedure, compared with SG and LAGB, Insufficient secretion of endogenous factors due to reduced gastric capacity and intestinal rearrangement, resulting in reduced absorption of vitamin B12, so we recommend postoperative supplementation and long-term, regular laboratory monitoring should have adhered to (42).

The duodenum and proximal jejunum are the best sites for dietary calcium absorption, where vitamin D is involved. Therefore, calcium deficiency can be further exacerbated if the diet is deficient in vitamin D (43). Food does not pass through the duodenum after RYGB, bypassing these parts of the small intestine, which reduces calcium and vitamin D intake, ultimately leading to calcium deficiency (44). Bone mineral density typically decreases after BS due to altered mechanical loading of the bone, whereas parathyroid hormone production increases. If parathyroid hormone continues to rise and hyperthyroidism occurs, it can lead to osteopenia and osteoporosis. Therefore, metabolic bone disease is a long-term risk factor after BS bypass surgery (45). In contrast, SG and LAGB did not alter duodenal traits, so calcium and magnesium deficiencies were less frequent.

After RYGB, iron deficiency was significantly higher in people with obesity than with SG and LAGB (46). This is because iron needs to be absorbed in the duodenum and proximal jejunum, with the participation of gastric acid (47). Divalent iron is better absorbed than trivalent iron when supplementing with iron after surgery (48). This is because ferrous iron is usually found in animal foods, while ferric iron is found in plant foods (49). Ferric iron needs to be reduced to ferrous iron under the action of gastric acid before it can be absorbed by the intestinal tract (50). Therefore, it is recommended to consume animal food after BS. RYGB is also a procedure with a high rate of vitamin D deficiency because it bypasses part of the duodenum and ileum, which is the main site of vitamin D absorption (51). RYGB is more likely to cause vitamin D deficiency than SG, while the defect in LAGB is not significant. For people with obesity, vitamin D deficiency should be detected early and supplemented in time to avoid the deterioration of the disease.

Regardless of the type of BS, it is inevitable that nutritional deficiencies will result. Our primary goals are to find the most appropriate procedure for people with obesity, minimize complications and nutritional losses, and monitor and supplement postoperatively. When selecting a procedure, people with obesity and doctor must also consider specific differences in postoperative nutritional needs and care (52).

In this study, we found that nutritional deficiencies after BS are widespread, although data from large, robust long-term RCTs are lacking. More studies are needed, such as direct comparisons of the effects of different BS procedures on clinically relevant nutritional deficiencies during the long-term, follow-up-especially for newer procedure types. Studying postoperative nutritional deficiencies in different subgroups (e.g., those with other diseases, special populations, or weaker social determinants of health) of BS should also be a high research priority.

### The limitation

Because our goal was to compare nutritional deficiencies after three commonly used bariatric surgeries at the same time (which can only be accomplished by constructing a network meta-analysis), we included surgical options in our network meta-analysis to help clinicians reduce the incidence of nutritional deficiencies in patients. We included all studies that fulfilled the inclusion criterion for the absence of risk. Small studies, to be sure, can introduce a lot of bias since there is greater uncertainty in the estimations and the outcomes are more likely to be random.

However, the quantity and quality of the identified evidence limit the strength of our conclusions. Only a few studies had sufficiently long follow-ups; however, we also note that the double-blind requirement may have artificially reduced the quality of the studies, which is rarely performed in trials evaluating surgical interventions. In addition, only a few studies reported patient undernutrition at baseline, but we attempted to address this limitation by performing a sensitivity analysis of these studies. Because this was an interim sensitivity analysis, the results should be interpreted cautiously. We included this part of the study in the heterogeneity study. For this study, we first noticed that there was no heterogeneity in these trials (*I*^2 ^= 0%) and that patients had nearly the same rate of the nutritional deficit as the group reporting baseline values. Secondly, In these trials, the follow-up time was so extensive that researchers may have thought that baseline levels could be ignored during long-term follow-up. As a result of these considerations, we believe that incorporating these trials in the network meta-analysis would have no detrimental consequences.

We followed PRISMA, developed a review protocol in advance, and mapped Brooks-Gelman-Rubin diagnostics charts. We used Mesh, a comprehensive literature search strategy, quality assessment and data extraction by repeat reviewers, and rigorous statistical methods to reduce potential bias. Given that the medical tools used to perform BS have advanced over time, there is very little literature on direct comparisons between different surgical approaches. However, we continue to use state-of-the-art, rigorous natural comparison methods to estimate the relative effectiveness of each procedure compared to others. Therefore, we are confident that our results are valid.

## Conclusion

Our network meta-analysis comparing the three most commonly used Bariatric surgery procedures yielded three key conclusions. First, high-quality data from randomized controlled trials remain insufficient—especially on calcium and vitamin D deficiencies after Bariatric surgery. Second, RYGB appears to be more likely to cause nutritional deficiencies after Bariatric surgery. Third, we determined the consequences of nutritional deficiencies between different surgical procedures. Although the nutritional deficiency after RYGB is slightly more severe than the other two procedures (i.e., SG and LAGB), it is still the most commonly used bariatric surgery in clinical practice.

This study suggests that choosing the three most common BS procedures is a trade-off between safety and efficacy. The information synthesized here can help people with obesity and physicians decide on the type of procedure to undergo or perform. We believe that the nutrient comparisons provided by our web-based meta-analysis can also help people with obesity and physicians understand the relative effectiveness of different procedures and incorporate them into their decision-making along with other essential data elements, e.g., local expertise and adverse (Effectiveness profiles).

## Data Availability

The original contributions presented in the study are included in the article/Supplementary Material, further inquiries can be directed to the corresponding author/s.
